# Older adults show biomarker evidence of PICS after sepsis

**DOI:** 10.1093/geroni/igab046.2528

**Published:** 2021-12-17

**Authors:** Robert Mankowski, Stephen Anton, Gabriela Ghita, Christiaan Leeuwenburgh, Lyle Moldawer, Philip Efron, Scott Brakenridge, Frederick Moore

**Affiliations:** 1 University of Florida, Gainesville, Florida, United States; 2 University of Florida, University of Florida, Florida, United States

## Abstract

Background: Hospital deaths after sepsis have decreased substantially and most young adult survivors rapidly recover (RAP). However, many older survivors develop chronic critical illness (CCI) with poor long-term outcomes. The etiology of CCI is multifactorial and the relative importance remains unclear. Sepsis is caused by a dysregulated immune response and biomarkers reflecting a persistent inflammation, immunosuppression and catabolism syndrome (PICS) have been observed in CCI after sepsis. Therefore, the purpose of this study was to compare serial PICS biomarkers in a) older (versus young) adults and b) older CCI (versus older RAP) patients to gain insight into underlying pathobiology of CCI in older adults. Methods: Prospective longitudinal study with young (≤ 45 years) and older (≥ 65 years) septic adults who were characterized by a) baseline predisposition, b) hospital outcomes, c) serial SOFA organ dysfunction scores over 14 days, d) Zubrod Performance status at three, six and 12-month follow-up and e) mortality over 12 months. Serial blood samples over 14 days were analyzed for selected biomarkers reflecting PICS. Results: Compared to the young, more older adults developed CCI (20% vs 42%) and had markedly worse serial SOFA scores, performance status and mortality over 12 months. Additionally, older (versus young) and older CCI (versus older RAP) patients had more persistent aberrations in biomarkers reflecting inflammation, immunosuppression, stress metabolism, lack of anabolism and anti-angiogenesis over 14 days after sepsis. Conclusion: Older (versus young) and older CCI (versus older RAP) patient subgroups demonstrate early biomarker evidence of the underlying pathobiology of PICS.

